# 206 MHz fully stabilized all-PM dispersion-managed figure-9 fiber laser comb

**DOI:** 10.1038/s41598-024-57735-2

**Published:** 2024-03-26

**Authors:** Shotaro Kitajima, Kwangyn Jung, Norihiko Nishizawa

**Affiliations:** Department of Electronics, Nagoya University, Furo-Cho, Chikusa-Ku, Nagoya, 464-8603 Japan

**Keywords:** Fibre lasers, Mode-locked lasers, Ultrafast lasers, Frequency combs

## Abstract

High-repetition-rate optical frequency combs are useful for precision spectroscopy because of their high power per comb mode, but conventional high-repetition-rate lasers do not have a broad enough spectrum. In this study, a fully stabilized polarization-maintaining figure-9 mode-locked fiber laser with a high repetition rate of 206 MHz and a broad spectrum was demonstrated by employing simultaneous control of cavity dispersion and length. The laser exhibited a 3 dB spectral bandwidth of 88 nm and a compressed pulse width of 66 fs. Additionally, *f*_CEO_ and *f*_rep_ phase locking were implemented, resulting in low (0.21 rad) in-loop carrier-envelope-offset frequency phase noise. To the best of our knowledge, this is the widest spectrum bandwidth and shortest pulse duration directly obtained from an all-PM figure-9 fiber laser oscillator to date. The combination of high repetition rate and broad spectral range makes this system very useful for a wide range of applications, especially in the field of precision spectroscopy.

## Introduction

Ultrashort-pulse fiber lasers with high repetition rate and short pulse duration have played a vital role in many applications, such as frequency metrology^1^, spectroscopy^2^, and microwave generation^3^. Especially in the field of optical frequency combs and their applications^4,5^, a high repetition rate is strongly desired to increase the power per comb mode and to improve the SNR of the beat signal^6^.

The highest repetition rate in fiber lasers can be obtained with a linear cavity mode-locked laser using a material-based saturable absorber (SA). A semiconductor saturable absorber (SESAM), nano-carbon material (SWCNT, Graphene), 2D material (TMDC), etc. have been reported as SAs for fiber lasers so far. In a linear cavity with an SA, a wavelength division multiplexer (WDM) for pump combining can be placed outside the cavity, and the cavity can be formed with only gain fiber and an SA, making it easy to achieve a fundamental repetition rate in the GHz range^7,8^. However, material saturable absorbers limit pulse widths due to their slow response time, shallow modulation depth, and large non-saturation absorption losses. Wide pulse widths result in larger intrinsic phase noise of *f*_CEO_ and timing jitter, which are undesirable for optical frequency combs. On the other hand, artificial saturable absorbers are the most suitable form for low-noise optical frequency combs because they provide deep modulation depth and fast response speed with low loss. The most common type of artificial saturable absorber for fiber lasers is nonlinear polarization evolution (NPR)^9^. In the high-repetition-rate region, so far, NPR fiber lasers have been realized at a repetition rate of 225 MHz, a spectral bandwidth of 135 nm, and a pulse duration of 37.4 fs^10^. An NPR fiber laser can achieve both low noise characteristics and a broad spectrum, but it is inherently sensitive to environmental changes because it cannot be configured to maintain polarization and requires daily alignment.

A nonlinear amplified loop mirror (NALM)-based ultrashort pulsed fiber laser^11–13^ can achieve low noise performance and broad spectra, like an NPR fiber laser, and can be designed with an all polarization maintaining (PM) configuration and self-start capability, making it an ideal base light source for a fiber comb. However, since an NALM laser has more components than an NPR laser, there is a limit to the degree to which the cavity can be shortened. Shortening the cavity also makes it difficult to adjust the cavity net dispersion value, making it difficult to achieve the combination of a high repetition rate and a broad spectrum. There are two types of NALM, known as figure-8 and figure-9, depending on whether a loop mirror is used for transmission or reflection. The figure-9 type is more advantageous for shortening the cavity because it requires only one loop. An all-PM, figure-9 fiber laser with the highest yet reported repetition rate of 250 MHz and a spectral width of 43 nm has been developed^14^. Yi et al. reported an environmentally stable figure-9 fiber laser with a repetition rate of 199.6 MHz, a 3 dB spectral width of 78 nm, and a pulse width of 77 fs^15^. This is the shortest pulse width ever reported directly from an NALM mode-locked laser. However, only one case of a fully-stabilized optical frequency comb based on an NALM fiber laser with a repetition rate exceeding 200 MHz has been reported, although no details of the laser design and optical characteristics were provided^16^.

In this work, we developed a stretched-pulse mode-locked Er-fiber laser with a repetition rate of 206 MHz and a 3 dB spectral width of 88 nm, by simultaneously shortening the cavity length and controlling the dispersion in a figure-9 Er-doped fiber laser. Also, a compressed pulse duration of as short as 66 fs was achieved. This spectral bandwidth and pulse duration are record values for all PM figure-9 fiber lasers, to the best of our knowledge. Moreover, by detecting and stabilizing *f*_CEO_ and *f*_rep_ of the oscillator, we constructed a fully stabilized optical frequency comb, and thanks to the characteristics of the NALM fiber laser and its broadband spectrum, the measured in-loop carrier-envelope-offset frequency SSB phase noise was suppressed to only 0.208 rad.

## Experimental setup

### Figure-9 mode-locked fiber laser

**Figure 1:**
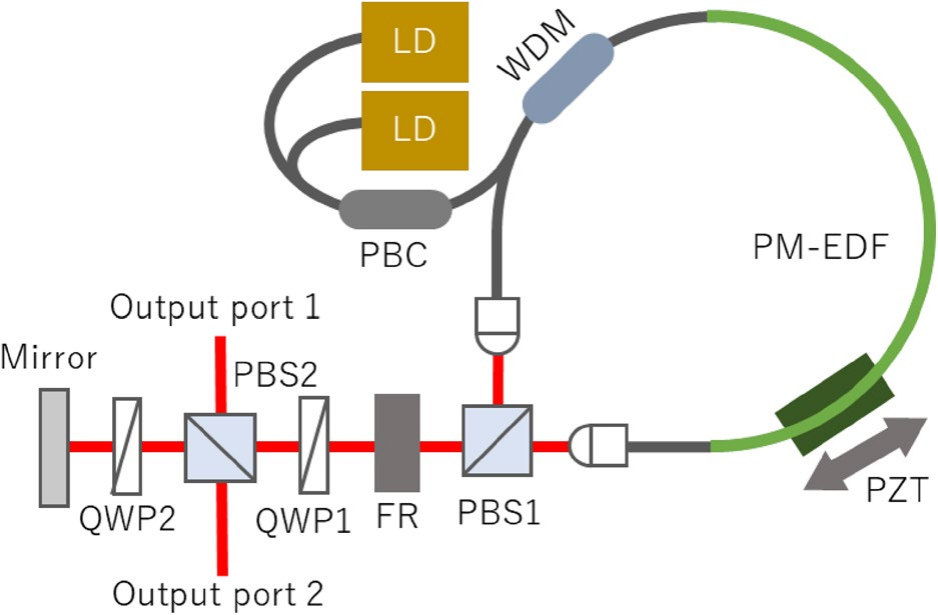
Setup of the all-PM figure-9 fiber laser oscillator. *LD* laser diode, *PBC* fiber type polarization beam combiner, *WDM* wavelength division multiplexer, *PM-EDF* polarization maintain Erbium-doped fiber, *PZT* piezoelectric actuator, *PBS* cube type polarization beam splitter, *FR* Faraday rotator, *QWP* quarter wave plate.

Figure 1 shows a schematic diagram of the 206 MHz figure-9 Er-doped fiber laser oscillator. A 50 cm long section of PM erbium-doped fiber (PM-EDF; iXblue IXF-EDF-HD-PM) was used as a gain fiber. The fiber section other than the EDF was common PM-SMF, with a total length of 36 cm. The normal dispersion of the PM-EDF canceled out the anomalous dispersion of the PM-SMF, and the calculated net cavity dispersion value was + 0.0039 ps^2^, which was small enough to obtain stretch pulse mode-locking. As a pump source, two fiber-coupled laser diodes (LDs) with a total maximum output power of 1.5 W and a center wavelength of 980 nm were used. The two collimators at the loop exit were rotated 90° to each other, and the output s- and p-polarized light components were combined at the polarization beam splitter/combiner (PBS). The free space section contained two PBSs, a Faraday rotator (FR), and two quarter-wave plates (QWP1 and QWP2). The modulation depth of the cavity could be optimized by adjusting the angle of QWP1. There were two output ports, called output port 1 and output port 2. The coupling ratio of output port 2, as well as the linear loss in the cavity, could be changed arbitrarily by adjusting the angle of QWP2. A PZT actuator was bonded to part of the PM-EDF to control the cavity length. First, the PZT was attached to the back side of the end mirror, but this caused too much crosstalk to *f*_CEO_, so it was then bonded to the fiber. The PZT connection to the back of the end mirror affected not only the cavity length but also the angle of the end mirror, resulting in an increase in linear loss in the cavity, which had a significant effect on *f*_CEO_. The entire optical system was built on an air-floating optical table to eliminate the effects of vibrations from the ground. Also, the laser cavity was temperature-stabilized with a water-cooled breadboard and covered by an acrylic enclosure with sound-absorbing sponges to suppress acoustic noise. The output pulse was analyzed with an autocorrelator (Femtochrome FR-103XL), an optical spectrum analyzer (Yokogawa, AQ6375B), and an RF spectral analyzer (Anritsu MS2840A). The output pulse was compressed by passing it through a dispersion compensation fiber (DCF) with a length of 55 cm and a β_2_ of 79 ps^2^/km. The compressed pulse was analyzed with custom-made second harmonic frequency-resolved optical gating (SHG -FROG).

### Fully stabilized optical frequency comb system

**Figure 2:**
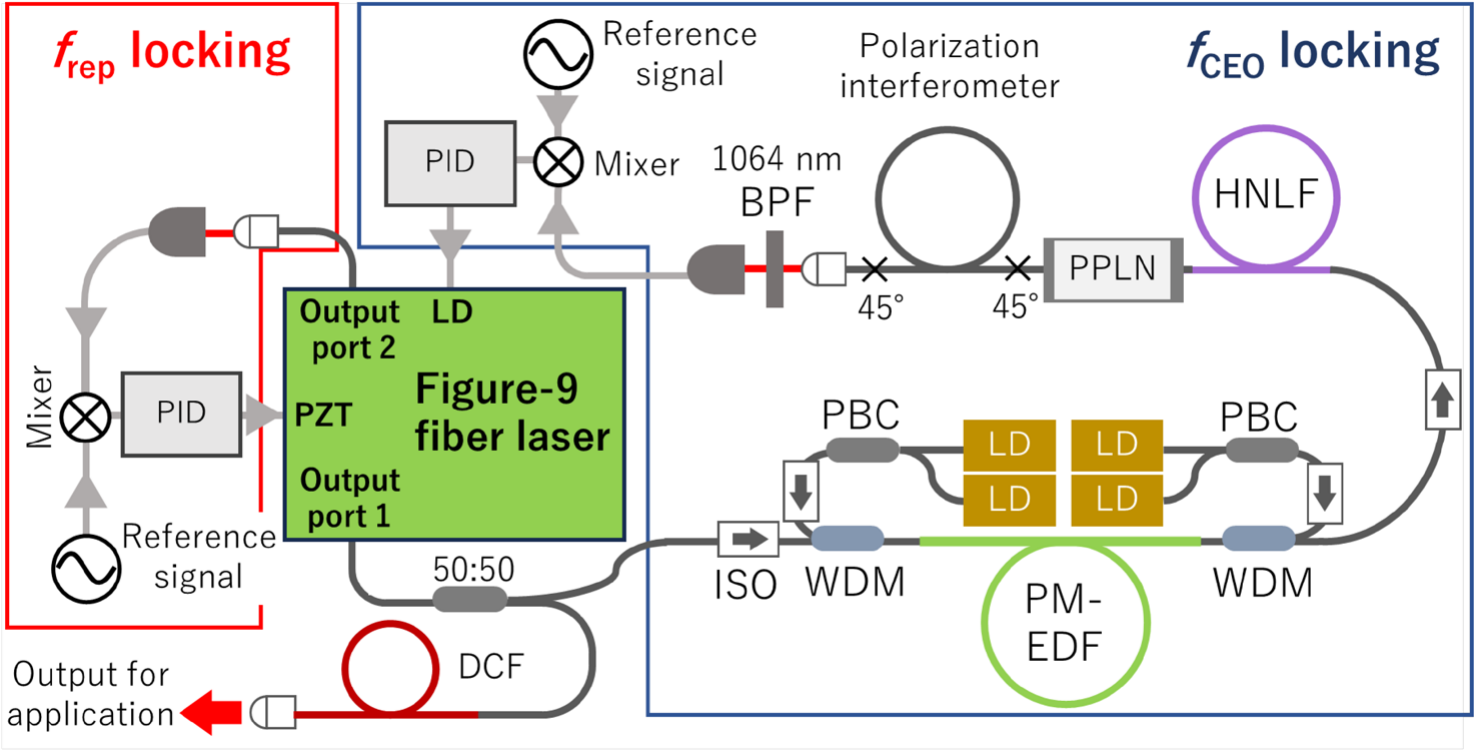
Setup of all-PMfigure-9 fiber laser-based fully stabilized optical frequency comb system. *PID* PID servo circuit, *BPF* band pass filter, *ISO* isolator, *PBC* fiber-type polarization beam combiner, *HNLF* high nonlinear fiber, *PPLN* in-line type periodically poled lithium niobate second harmonic generator, *DCF* dispersion compensation fiber.

Figure 2 shows the whole setup of the optical frequency comb system, including an oscillator, *f*_CEO_ detection optics, and phase stabilizing circuits. Of the two output ports, the output of port 1 was divided by a 50:50 coupler, and one output was used for *f*_CEO_ detection, whereas the other one was used for applications. The output of port 2 was used for optical spectrum monitoring and *f*_rep_ detection. The output power of the application port was 12 mW. *f*_CEO_ detection was performed using the *f*-2f. interferometry method. The *f*_CEO_ stabilization optics consisted of a PM-EDF amplifier, a highly nonlinear fiber (HNLF), a waveguide-type periodically poled lithium niobate (PPLN) second harmonic generator, and a polarization interferometer for temporal delay adjustment^17^. Phase detection circuits for *f*_rep_ and *f*_CEO_ consisted of a band pass filter (BPF), a low-noise RF amplifier, and a frequency mixer. As reference signals, a signal generator (Agilent 8648C) and a function generator (Tektronix AFG1062) were used for *f*_rep_ and *f*_CEO_, respectively. The generated *f*_CEO_ error signal was fed back to the oscillator LD current through a PID circuit (Vescent D2-125). The *f*_rep_ signal was fed back to the PZT in the cavity through another PID circuit (Turtle industry, T-PID01Z). Both of the stabilized frequencies were analyzed with an RF spectral analyzer (Anritsu MS2840A) and an FFT analyzer (Stanford Research Systems SR770).

## Results

### Output properties of figure-9 mode-locked fiber laser

**Figure 3:**
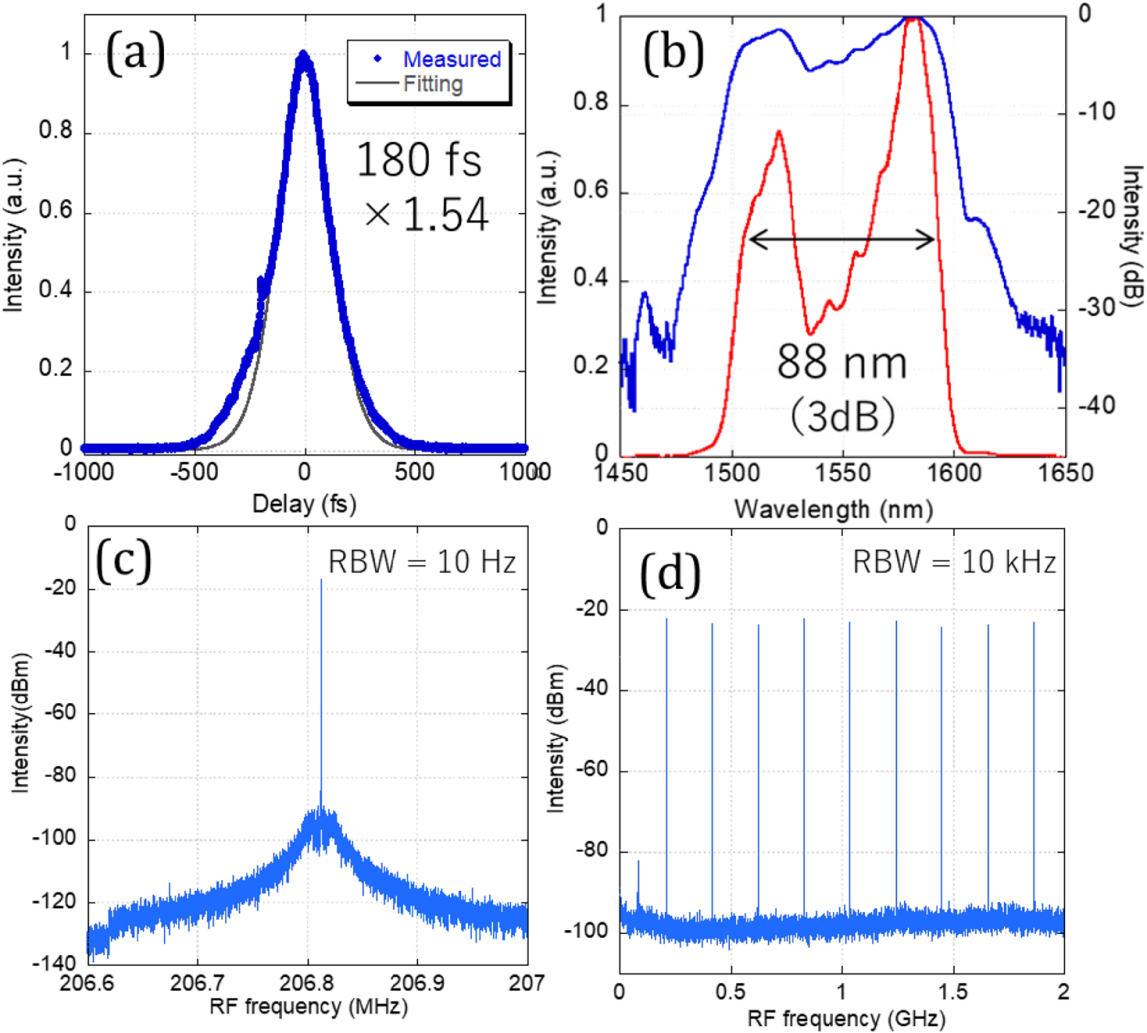
Output characteristics of all-PM figure-9 fiber laser, (**a**) autocorrelation trace, (**b**) output spectra (blue line: log scale, red line: linear scale), (**c**) RF spectra of fundamental beat note, (**d**) RF spectrum of higher order beat notes up to 2 GHz.

The mode-locking was self-started by increasing the total output power of the two LDs to about 1.4 W. At this time, there was a CW peak in the spectrum, but when the pump power was reduced to about 1.0 W, the state changed to a single-pulse mode-locked state with no CW peaks. The angle of the two QWPs inside the cavity greatly affected the mode-locking condition, but once set to the optimum values, the waveplates did not need to be touched again to initiate mode-locking. Figure 3 shows the oscillation spectrum, autocorrelation trace, and radio frequency (RF) spectrum of the mode-locked output from output port 1. The average output power was about 30 mW from output port 1 and 0.5 mW from output port 2 when the pump power was 0.80 W. The measured pulse duration was 180 fs (assuming a Gaussian shape) (Fig. 3a), which was broadened at the output fiber. The spectrum showed the typical shape of stretched-pulse mode locking, and a 3 dB bandwidth of 88 nm was obtained, although the center of the spectrum was depressed (Fig. 3b). The RF spectra (Fig. 3c,d) showed SNRs above 70 dB and equally spaced beat notes of uniform amplitude, indicating that the oscillator was in a clean single-pulse mode-locking state. The repetition rate was variable between 205 and 209 MHz by adjusting a motor stage at the end mirror.

**Figure 4:**
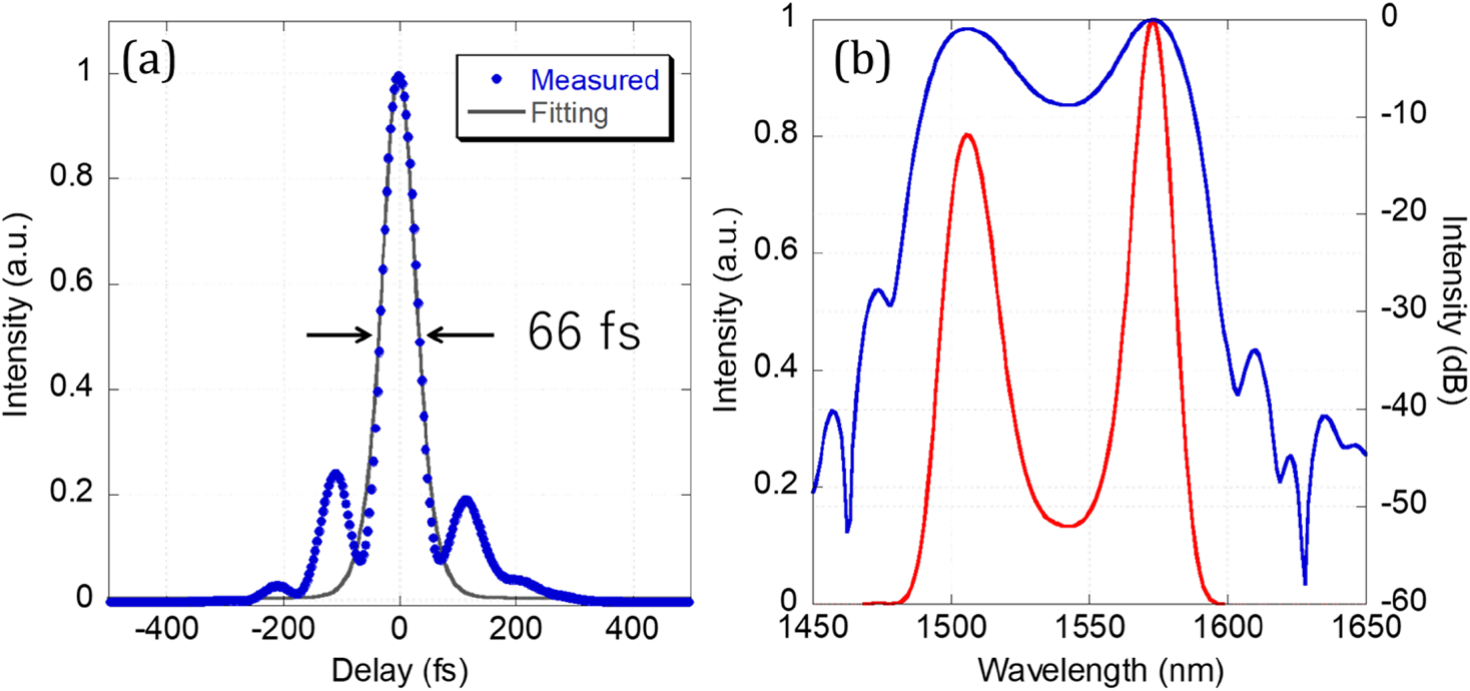
(**a**) Pulse shape and (**b**) optical spectrum (blue line: log scale, red line: linear scale) of compressed output pulse measured by SHG FROG.

Since the output pulses were mainly broadened by the patch fiber after output and had a linear chirp, they could be easily compressed by a DCF with normal dispersion. Figure 4 shows the temporal waveform and spectrum of the compressed pulse measured by SHG FROG, with a FROG error of 0.58%. The measured pulse duration was as short as 66 fs. To the best of our knowledge, this is the shortest pulse duration directly obtained from an NALM fiber laser. However, the transform-limited pulse duration calculated from the spectrum is 31 fs, which suggests that further shortening of the pulse is possible by compensating for higher-order dispersion and eliminating the additional nonlinearity in the DCF.

### Properties of fully stabilized optical frequency comb

**Figure 5:**
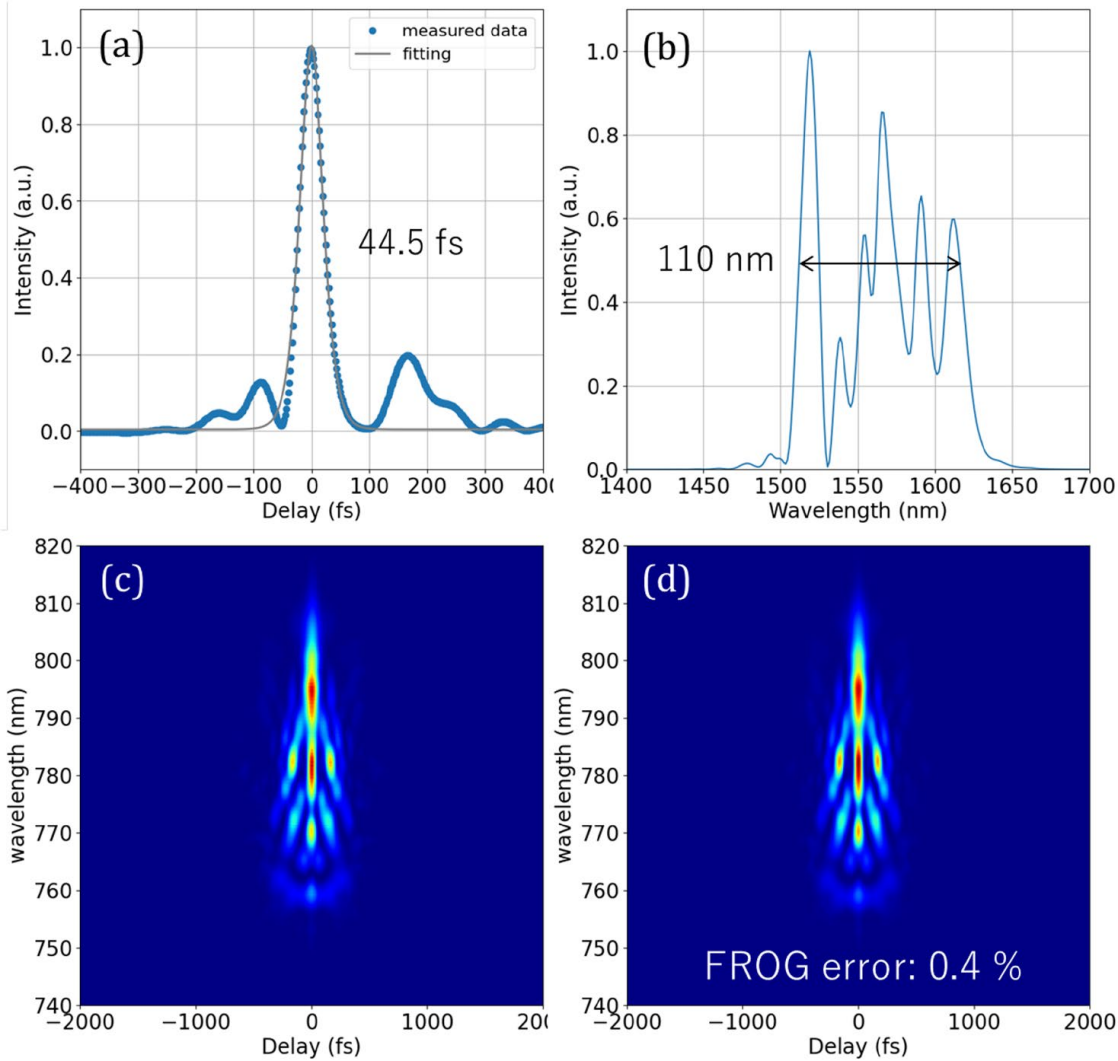
Characteristics of amplified pulses after EDFA measured by SHG FROG: (**a**) pulse shape, (**b**) output spectra, (**c**) measured FROG spectrogram, and (**d**) reconstructed FROG spectrogram.

**Figure 6:**
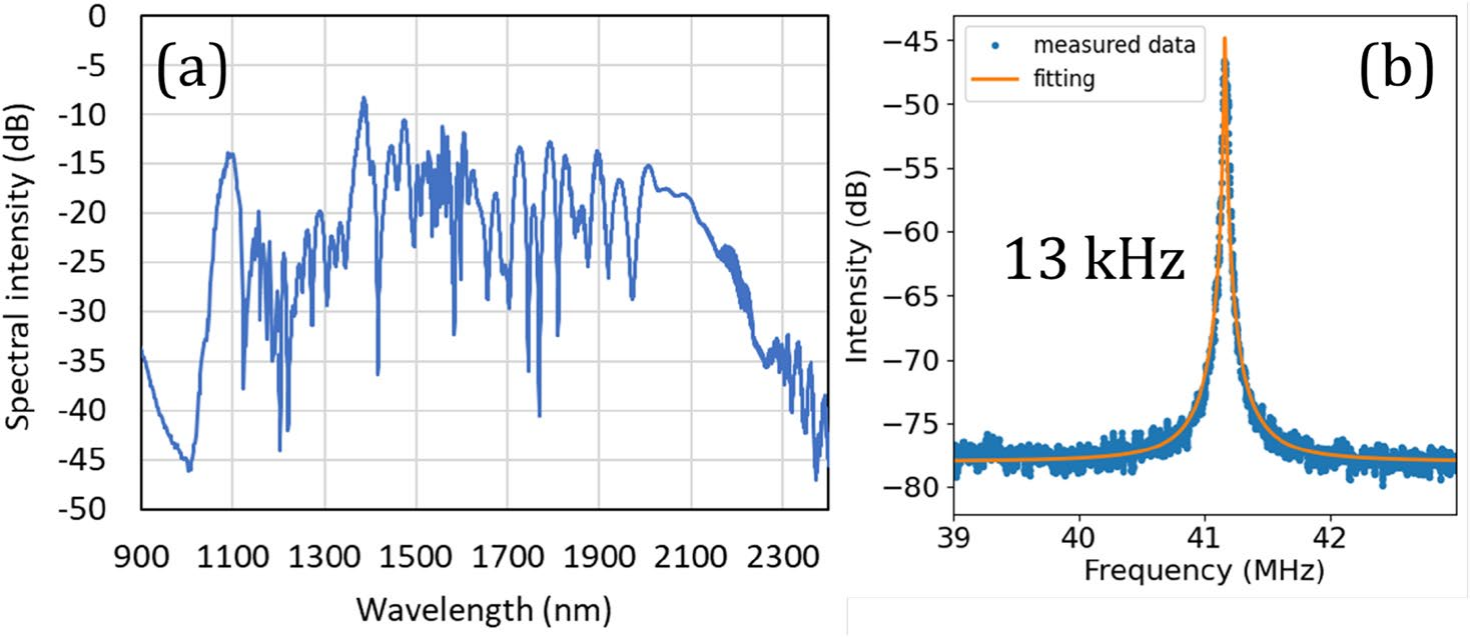
(**a**) Octave spanning supercontinuum spectrum after HNLF. (**b**) RF signal of free-run f_CEO_. The orange line shows a squared-Lorentzian fitting.

Figure 5 shows the spectrum and temporal pulse shape of the amplified output pulses measured by SHG-FROG. The spectrum was expanded to 110 nm due to the similariton effect during EDFA propagation. The pulses were optimally dispersion compensated by the cutback method, and the average output power, pulse duration, and peak power of the pulses were measured to be 440 mW, 44.5 fs, and 45 kW, respectively. By using 20 cm HNLF (γ = 21 W^-1^ km^-1^, β_2_ = 0.25 ps^2^/km), an octave-spanning spectrum from 1064 to 2128 nm was obtained (Fig. 6a). The *f*_CEO_ signal was detected by a balanced detector by generating a second harmonic through a waveguide PPLN (NTT Electronics Corp., WH-1064) and using a polarization interferometer to adjust the time interval between the fundamental and SHG signals. Figure 6b shows the free-running *f*_CEO_ signal. A free-run linewidth of 13 kHz and an SNR of 33 dB were obtained by Lorentz function fitting. The position of the *f*_CEO_ can be adjusted from 3 to 100 MHz by pump LD current control. For the phase locking, a range of 20–40 MHz was selected to obtain the highest SNR.

**Figure 7:**
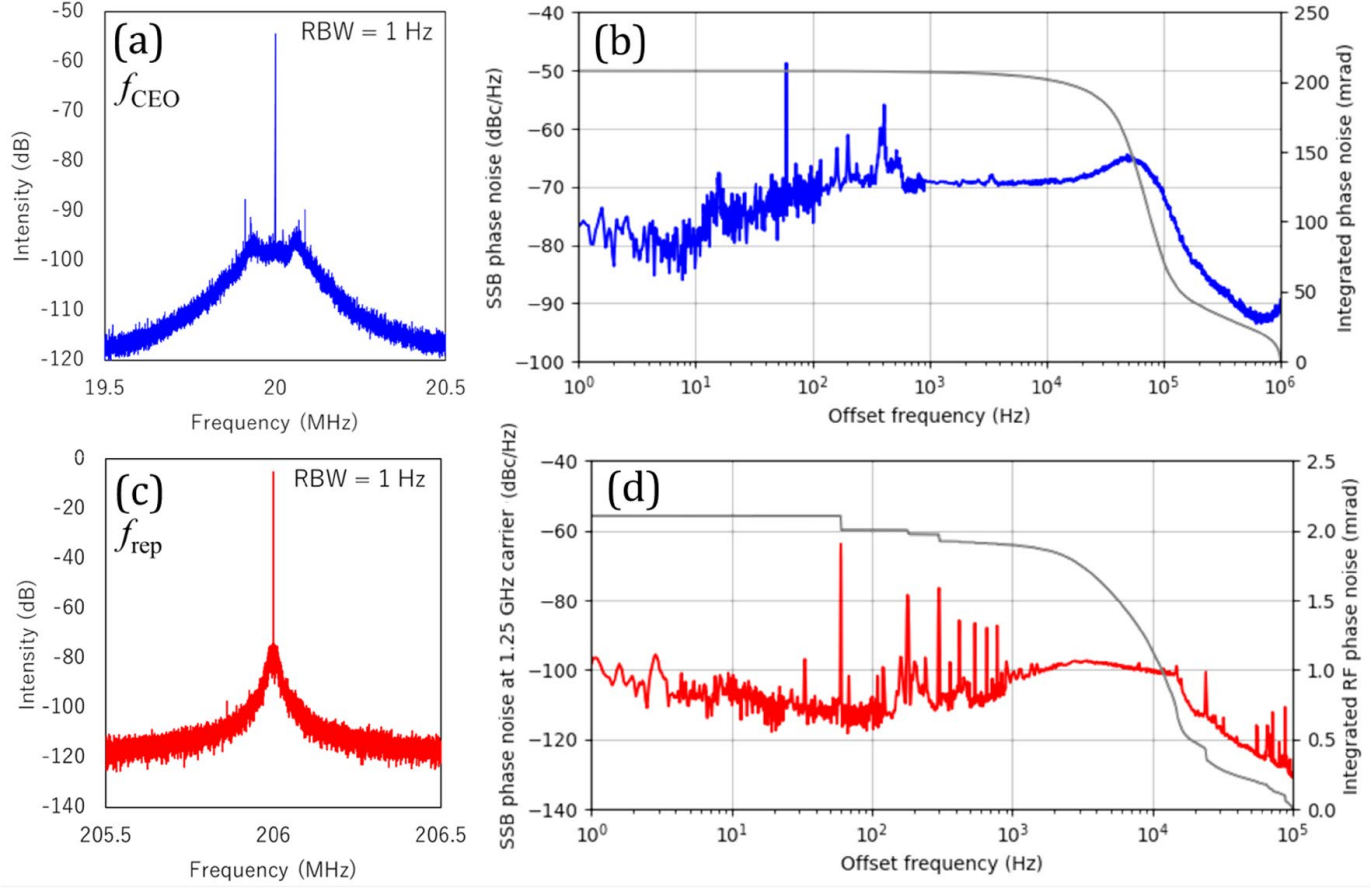
(**a**) RF spectrum of phase-locked *f*_CEO_ with 1 Hz RBW. (**b**) In-loop SSB phase noise and integrated phase noise of phase-locked *f*_CEO_. (**c**) RF spectrum of phase-locked *f*_rep_ with 1 Hz RBW. (**d**) In-loop SSB phase noise and integrated phase noise of phase-locked *f*_rep_.

Figure 7 shows the RF spectra and phase noise of the stabilized *f*_CEO_ and *f*_rep_. The measured integrated phase noise of *f*_CEO_ was 0.208 rad (1 Hz to 1 MHz), indicating that low-noise stabilization was achieved. The integrated timing jitter was calculated to be 0.17 fs. The control bandwidth of *f*_CEO_ estimated from the servo bump was about 50 kHz, which was limited by the slow response of the pump modulation. By increasing the P-gain (*K*p), the control bandwidth could be extended to about 150 kHz, but the integrated phase noise was degraded due to the large servo bump caused by the reduced phase margin. Additional fast-loss modulation, such as a graphene modulator, can be used to achieve a wider control bandwidth and lower noise^18^. The integrated phase noise of *f*_rep_ was 2.1 mrad. However, in the case of locking to the RF reference, the phase noise is multiplied by the number of comb modes, *N* (≈ 10^6^), so it is necessary to use a beat signal with an optical frequency reference such as a cavity-stabilized CW laser instead of an RF reference to achieve more stable phase locking. A long-term operation test of the fully stabilized comb, including continuous measurements of *f*_CEO_ and *f*_rep_ using a frequency counter, was also conducted for one hour. The gate time of the frequency counter was set to 1 s. Standard deviations for *f*_CEO_ and *f*_rep_ were estimated to be about 690 μHz, and 343 μHz, respectively. We confirmed the phase-locking operation for several hours, and thanks to the shielding and temperature control, drift in both signals during free running was minimal, and phase locking never dropped unless unexpected large vibrations or disturbances occurred.

## Discussion and conclusion

Table 1 shows a comparison of the performance of NALM Er-doped fiber lasers with repetition rates above 200 MHz^14,15,19,20^. The spectral bandwidth obtained in this study is the highest yet reported. This is also the second report of a fully stabilized NALM Er-doped fiber laser in an all-PM configuration with a high repetition rate. Ref. 19 reported the highest repetition rate and the shortest pulse duration by using a special optical component, but the fiber laser reported there was not an all-PM configuration, and the spectrum had a large modulation. To further enhance the repetition rate, it is necessary to reduce the length of the fiber section. The fibers utilized in this study include PM-EDF with normal dispersion and PM-SMF with anomalous dispersion. Their lengths were adjusted to approach zero dispersion. The primary limiting factor is the length of the EDF, which proves challenging to significantly shorten while maintaining sufficient gain. If a higher concentration PM-EDF were available, it is anticipated that further shortening of the cavity could be achieved. The free-space section has already been reduced to approximately 10 cm in this study, with additional shortening yielding minimal impact. Practical limitations also arise from the fusion splicer. The fusion splicer employed in this study necessitates a minimum length of about 7 cm at both ends. The combined length of the two collimators and the pigtail fibers at both ends of the WDM is a minimum of approximately 28 cm. To solve this problem, it is useful to directly connect the EDF to the WDM or package multiple elements, such as the collimator and WDM. Alternatively, placing the WDM outside the resonator presents another viable option.

**Table 1:** Comparison of NALM mode-locked Er fiber lasers with repetition rates above 200 MHz.

References	This work	^14^	^15^	^19^	^20^
Repetition rate [MHz]	206	250	200	201	257
Spectral bandwidth [nm]	88	43	78	21	-
Pulse duration [fs]	66	–	77	510	44.6
All-PM configuration	Yes	Yes	Yes	Yes	No
Fully stabilized	Yes	Yes	No	No	No

In conclusion, we report the successful development of a fully-stabilized polarization-maintaining figure-9 fiber mode-locked laser comb with a repetition rate of 206 MHz and a wide spectrum. The 3 dB spectral bandwidth and compressed pulse width achieved were 88 nm and 66 fs, respectively. To the best of our knowledge, this represents the widest spectral bandwidth and shortest pulse duration directly obtained from an all-PM figure-9 laser oscillator. Additionally, phase locking of *f*_CEO_ and *f*_rep_ was performed, resulting in low (0.21 rad) carrier-envelope-offset frequency phase noise. The significance of this work lies in the achievement of a high repetition rate and short pulse duration, which are crucial for applications such as frequency metrology, spectroscopy, and microwave generation. Using methods such as spectrum filtering with nonlinear optics, it is possible to concentrate energy into a small number of comb modes. For example, a Fabry–Perot etalon^21^, VIPA^22^, and spectral peaking^23–25^ can be used. Even when employing such techniques, a wider comb spacing in the base light source is advantageous. Hence, we believe that lasers with high repetition rates and wide spectra, as demonstrated in this study, offer distinct advantages in various applications.
